# Detecting Malaria Hotspots in Haiti, a Low-Transmission Setting

**DOI:** 10.4269/ajtmh.20-0465

**Published:** 2021-04-19

**Authors:** Amber M. Dismer, Jean Frantz Lemoine, Mérilien Jean Baptiste, Kimberly E. Mace, Daniel E. Impoinvil, Jodi Vanden Eng, Michelle A. Chang

**Affiliations:** 1 Centers for Disease Control and Prevention, Division of Global Health Protection, Atlanta, Georgia;; 2 Ministry of Public Health and Population, National Malaria Control Program, Port-au-Prince, Haiti;; 3 Centers for Disease Control and Prevention, Division of Parasitic Disease and Malaria, Atlanta, Georgia

## Abstract

In 2006, Haiti committed to malaria elimination when the transmission was thought to be low, but before robust national parasite prevalence estimates were available. In 2011, the first national population-based survey confirmed the national malaria parasite prevalence was < 1%. In both 2014 and 2015, Haiti reported approximately 17,000 malaria cases identified passively at health facilities. To detect malaria transmission hotspots for targeting interventions, the National Malaria Control Program (NMCP) piloted an enhanced geographic information surveillance system in three departments with relatively high-, medium-, and low-transmission areas. From October 2014–September 2015, NMCP staff abstracted health facility records of confirmed malaria cases from 59 health facilities and geo-located patients’ households. Household locations were aggregated to 1-km^2^ grid cells to calculate cumulative incidence rates (CIRs) per 1,000 persons. Spatial clustering of CIRs were tested using Getis-Ord Gi* analysis. Space–time permutation models searched for clusters up to 6 km in distance using a 1-month malaria transmission window. Of the 2,462 confirmed cases identified from health facility records, 58% were geo-located. Getis-Ord Gi* analysis identified 43 1-km^2^ hotspots in coastal and inland areas that overlapped primarily with 13 space–time clusters (size: 0.26–2.97 km). This pilot describes the feasibility of detecting malaria hotspots in resource-poor settings. More data from multiple years and serological household surveys are needed to assess completeness and hotspot stability. The NMCP can use these pilot methods and results to target foci investigations and malaria interventions more accurately.

## INTRODUCTION

Malaria is a debilitating disease and can result in death. In Haiti, malaria is caused primarily by *Plasmodium falciparum* and is transmitted by *Anopheles albimanus*.^1^ All other countries in the Caribbean region, besides Haiti and the Dominican Republic on the island of Hispaniola, have eliminated malaria. Joining the historic effort to create a malaria-free zone in the Caribbean, Haiti’s Ministry of Public Health and Population (MSPP) and National Malaria Control Program (NMCP) committed in 2006 to eliminating malaria; at that time, the target elimination year was 2020.^2^ In 2011, Haiti’s first national population-based survey for malaria documented the low parasite prevalence of < 1%, supporting the feasibility of malaria elimination.^3^ In 2011, Haiti’s NMCP reported 32,969 confirmed malaria cases detected by microscopy.^4^ By 2015, with the rollout of rapid diagnostic testing (RDT) in 2013 to 2014 and improved case management, confirmed malaria cases declined to 17,583.^4^

For countries approaching malaria elimination, the WHO recommends several steps to achieve elimination: When all departments achieve < 1% parasite prevalence or < 100 cases per 1,000 annual parasite incidence (API), countries should initiate case-based passive surveillance, generate individual line listings of confirmed cases detected at health facilities, geo-locate cases at the household level, conduct reactive case detection activities, and complete entomological investigations.^5,6^ The WHO advocates for malaria teams consisting of surveillance and entomological staff to delineate boundaries for malaria transmission by using 1) geo-located confirmed cases and 2) ecological information such as larval development sites.^6^ These delineated areas are defined as foci. The WHO emphasizes that to be considered a focus, the area must contain both “the epidemiological and the ecological factors necessary for malaria transmission.”^6^ In countries where there is focal and heterogeneous transmission, the WHO promotes the efficient use of available resources by targeting interventions at transmission foci.^6^

In Haiti, as in other countries working to eliminate malaria, there are many operational challenges to identifying an area as a malaria transmission focus. Foci investigations are time intensive, require both entomological and epidemiological staff, incur high travel costs and per diem funds, and have a lengthy administrative process to deploy staff outside the departmental capitals. With limited personnel and financial resources, Haiti’s NMCP used an alternative approach initially to identify hotspots of confirmed malaria cases. Bousema et al.^7^ define malaria hotspots as areas “where transmission intensity exceeds the average level,” writing that malaria hotspots are, on average, < 1 km^2^ in size and are often within a malaria focus. A stable hotspot promotes transmission across both dry and rainy seasons, so with surveillance activities, persistent hotspots could be detected across time.^7^ Identifying smaller hotspots (e.g., at the village or household level) and characterizing whether these hotspots persist across time may help determine the level and type of response, and the amount of resources that are warranted.

In this context, Haiti’s NMCP established a pilot geographic information system (GIS) enhanced surveillance system to detect malaria hotspots in three of the 10 departments. In 2015, the Grande Anse Department (population: 468,301) reported the highest number of malaria cases, > 40% of the country’s total, with an API of 14.9 cases per 1,000 persons. The Sud Department (population: 774,976) reported a medium number of malaria cases (7% of total; API of 1.5) and the Sud-Est Department (population: 632,601) noted a low number of cases (3% of total; API of 0.8), which highlighted the heterogeneity of malaria transmission across Haiti.^8^ Because these departments represented similar but varied malaria transmission profiles, the NMCP selected these three departments for the pilot.

The primary objective of the GIS enhanced surveillance system was to characterize the location of malaria hotspots and to determine cluster size and cluster duration in the Grande Anse, Sud, and Sud-Est departments. The pilot system identified test-confirmed malaria cases from health facilities and, retrospectively, geo-located the confirmed cases at their residences. Then, geospatial analyses using these household-level data were performed to detect malaria hotspots. The secondary objective of the pilot was to identify operational requirements, personnel, and travel resources needed, and the challenges encountered to provide information for expanded case-based reporting for future years. If successful, the identification of malaria hotspots would be invaluable for targeting interventions in Haiti.

## METHODS

### Pilot areas.

**Figure 1.**
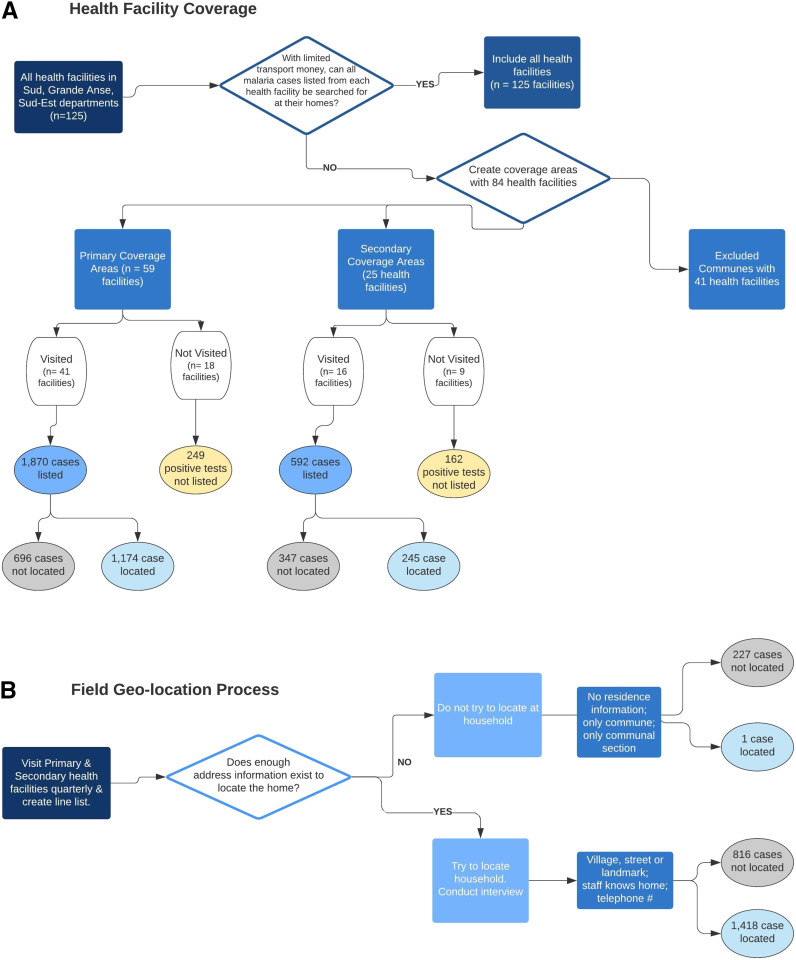
Pilot geographic information system enhanced surveillance system workflow and health facility inclusion criteria. (**A**) Health facilities in the Department were covered using the following criteria. (**B**) This is the process GIS Assistants used to locate patients at their homes and collect GPS coordinates. This figure appears in color at www.ajtmh.org.

**Figure 2.**
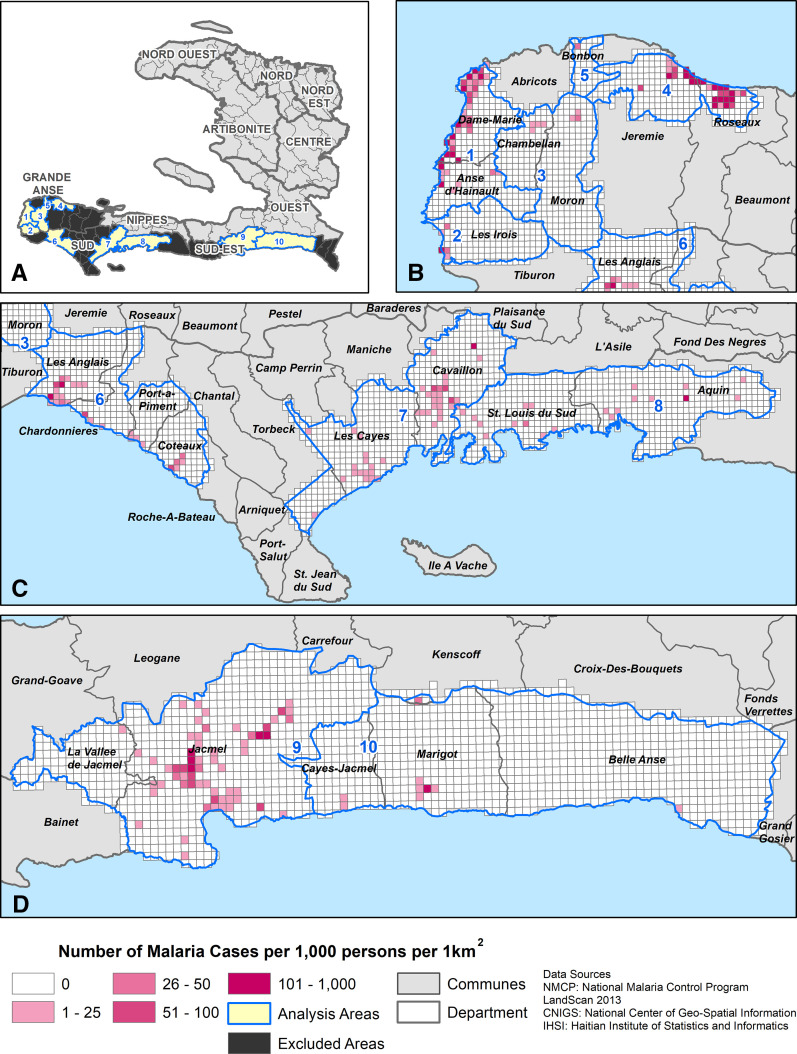
Adjusted cumulative incidence rate of malaria cases per 1,000 persons in Haiti. (**A**) Departments were split into primary (1, 4, 7, and 9) and secondary (2 and 3, 5 and 6, 8, and 10) coverage areas and excluded areas in black. Malaria cases were geo-located at their residences within coverage areas. (**B**) Cumulative incidence rate (CIR) in the Grande Anse Department of geo-located cases. (**C**) CIR in the Sud Department of geo-located cases. (**D**) CIR in the Sud-Est Department of geo-located cases. This figure appears in color at www.ajtmh.org.

The NMCP established a pilot GIS enhanced surveillance system to collect information retrospectively on patients with malaria diagnosed in health facilities from October 2014–September 2015. Each of Haiti’s 10 administrative department consists of communes; communes are further divided into communal sections (mean size: 48 km^2^, range: 3–278 km^2^).^8^ Three departments (Grande Anse, Sud, and Sud-Est) with the greatest number of reported cases in Haiti were selected for the pilot. Four GIS assistants conducting the field work resided in the following departments: Grande Anse (n = 2), Sud (n = 1), and Sud-Est (n = 1). One GIS analyst managed the program from Port-au-Prince. Initially, all communes in each department were included. Subsequently, in February 2015, the pilot area was reduced using travel costs, time, and malaria cases as the criteria (Figures 1 and 2A). Communes highlighted in black were excluded; the remaining communes were grouped into 10 coverage areas (indicated in blue in the online version of Figure 2A) designated as primary and secondary coverage areas.

### Health facility record review.

Because Haiti’s address hierarchy is limited, the NMCP could not link remotely case addresses collected at the health facility to a village data set. Standardized village boundaries do not exist, and villages can cross communal sections. Therefore, each case needed follow-up to identify the residence. GIS assistants obtained aggregate monthly numbers of confirmed malaria cases by health facilities from the national Health Surveillance Information System (HSIS). GIS assistants only visited health facilities with laboratory-confirmed malaria cases (RDT or microscopy positive). If a facility did not report any cases, it was not visited that quarter. Health facilities with greater numbers of confirmed malaria cases reported in the HSIS were visited first.

At each facility, GIS assistants examined clinical registers and created a line list of each confirmed case. To ensure completeness and avoid health facility recording anomalies, GIS assistants reviewed four data sources: 1) morbidity and mortality registers (MMRs), 2) emergency room registers, 3) the RDT registry, and 4) laboratory registers. Abstracted line lists included the patient’s name, guardian’s name (if younger than 18 years), age, gender, malaria test date, type of malaria test conducted, death status, telephone number, and address. Patients’ past medical charts were reviewed to obtain the village name and residential details (a street name, house number, or a community landmark) to search for the patient in the community. Because of the large geographic size of communal sections, patients whose records lacked a village name, a street name, or community landmark were not sought unless a phone number was recorded or staff members knew the residence (Figure 1).

### Geo-location and interview.

After compiling a list of individual laboratory-confirmed cases at the health facility, the GIS assistant traveled to locate the cases at their households. At the household, the GIS assistant acquired informed consent, then subsequently administered a short questionnaire to the patient, guardian, or proxy, and recorded responses on a paper form. The questionnaire confirmed each person’s age, gender, number of days with malaria symptoms, and whether the individual had traveled and slept at least one night in another commune in the previous month, and if so, the name of the visited commune. It also was used to collect data on the total number of persons living in the household from October 2014–September 2015 and the self-reported address. GPS coordinates and household elevation were captured at the front door. The GIS assistant did not conduct any malaria testing.

GIS assistants entered geo-located case questionnaires into a Microsoft Access^®^ database (Microsoft Corp., Redmond, WA) and sent the database to the GIS analyst within the NMCP. The database was cleaned and analyzed using Stata v. 12 (Stata Corp., College Station, TX), ArcGIS v. 10.2 (ESRI Inc., Redlands, CA), and SaTScan™ v. 9.4 (National Cancer Institute, Boston, MA).

### Geospatial analyses.

The LandScan 2013 world population data set was used to derive 1-km^2^ grids for Haiti.^9^ Cumulative incidence rates (CIRs) per 1,000 persons were created using aggregated confirmed cases per grid cell geo-located from October 2014–September 2015. CIRs were adjusted for areas with confirmed cases where LandScan predicted a population of zero; the adjusted population was an average of eight adjacent cells’ population.

The Average Nearest Neighbor tool in ArcGIS was used to calculate the average distance between households with confirmed malaria cases and to assess whether the households were clustered.^10^ Spatial autocorrelation was assessed using ArcGIS’s Global Moran’s Index (Moran’s I) at 1-km increments up to 6 km using the inverse distance relationship to examine whether there was a distance at which households with malaria were clustered. The maximum radius of 6 km corresponds with the reported maximum flight range of *A. albimanus* and was used for determining spatial autocorrelation and subsequent Getis-Ord Gi* analyses.^11,12^ The Global Moran’s I computes an expected index value and compares it with an observed index.^13^ The Incremental Global Moran’s I spatial autocorrelation provides the peak cluster distances that are considered significant and can be used in space–time models.^13^ The Getis-Ord Gi* analysis was conducted with the malaria CIR grid. This test uses the case locations and population distribution to identify clusters of malaria incidence for each coverage area.^14^

### Space–time permutation modeling.

A retrospective Kulldorff space–time permutation model was created for each coverage area using SaTScan v. 9.4 to search for hotspots containing high numbers of malaria cases with the minimum time aggregation at 1 month, according to a similar strategy used in previously reported research.^15–17^ A maximum time window of 6 months and a maximum radius of 3 km (slightly greater than the *Anopheles* average flight range) were used to detect clusters.^17^ No geographic overlap between the primary and secondary clusters was permitted. These models simulate points randomly inside the area in a cylindrical window; the simulation repeats 999 times using a Monte Carlo distribution and detects clusters that are significant across both space and time.^15^

### Ethics approval.

The malaria pilot GIS enhanced surveillance system was reviewed and approved by the Bioethics Committee of the Haitian MSPP, and the Office of the Associate Director of Science of the U.S. Centers for Disease Control and Prevention.

## RESULTS

### Confirmed cases from health facility reviews.

From October 2014–September 2015, 59 of 84 health facilities with confirmed malaria cases in the coverage zones were visited (Figure 1). In total, 2,462 confirmed cases were identified by chart reviews; 82.0% had been confirmed by the facilities via RDT only, 5.2% via microscopy only, and 12.8% via both methods. The use of two tests (RDT and microscopy) to confirm malaria for the same patient occurred at four health facilities.

Overall, 1,419 (58%) of 2,462 confirmed malaria cases were geo-located at their households (Table 1). Cases were located at the household between 1 week and 6 months after confirmation. A median of 16 cases were identified by chart review per health facility (range: 1–444 cases; interquartile range [IQR]: 41 cases).

**Table 1:** Line-listed confirmed cases identified in the field by geographic information system assistant

Coverage areas	GIS assistants (*N*)	Health facilities covered (*N*)	Commune population of primary and secondary coverage areas (*N*)†	Geographic area (km^2^)	Facility, line listing			Facility-based malaria testing		Community
					Cases (*N*)	Households (*N*)	Average no. of cases per household	Positive microscopy (*N*)	Positive RDT (*N*)	Field geo-located cases*: N* (% cases listed)
Grande Anse	2	27	326,211	768.7	1,685	1,460	1.15	327	1,659	972 (58)
Dame Marie*	1	14	156,138	578.0	1,148	1,051	1.09	301	1,148	493 (43)
Jérémie*	1	13	170,073	190.7	537	410	1.31	26	511	479 (89)
Sud	1	12	540,961	1,172.5	457	448	1.02	109	361	228 (50)
Sud-Est	1	20	414,679	1,183.2	320	278	1.15	6	314	219 (68)
Total	4	59	1,281,851	3,124.4	2,462	2,186	1.13	442	2,334	1,419 (58)

GIS = geographic information system; RDT = rapid diagnostic test.

* Dame Marie and Jérémie are two primary coverage areas within the Grande Anse Department, where two GIS assistants lived.

† Population is from the Haitian Institute of Statistics and Information in 2015.^8^

Of the 1,043 cases that were not geo-located at their households, GIS assistants did not search for 227 cases because their records lacked a village, street name, or landmark (Figure 1). The remaining 816 cases were not found. This may have been a result of the village being unknown, distant from agent’s post, and in mountainous terrain, or a result of insufficient travel funds or difficulty locating children younger than 18 years old because their names were not well-known in the community (*N* = 74 cases).

The median age of malaria cases was 17 years (range: < 1–97 years; IQR: 27 years) and the median number of days with symptoms before care-seeking was 2 days (range: 0–32 days; IQR: 4 days). Chart reviews showed five malaria infections resulted in death during the monitoring period. Of these five, three were children younger than 6 years old and three were seen at one facility. Less than 1% of all geo-located cases reported they traveled (e.g., spent at least one night outside their commune of residence) during the month before seeking health care. Twenty-one additional residents in households with confirmed malaria patients self-reported having received a malaria diagnosis; however, their diagnosis was not confirmed by laboratory records at the health facilities.

**Figure 3.**
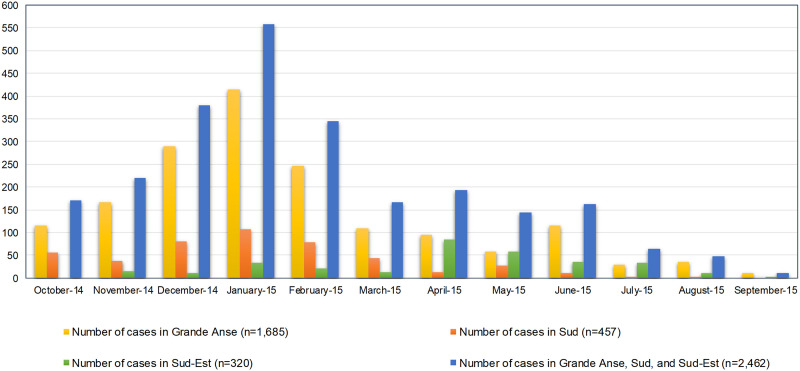
Line-listed confirmed cases at health facilities by month of diagnosis. This figure appears in color at www.ajtmh.org.

Overall, two seasonal peaks were detected: one large peak in January 2015 (*N* = 557 cases) for all departments and a second small peak in June 2015 (*N* = 116 cases) for the Grande Anse Department (Figure 3, epidemiology curve). From October 2014–March 2015, there were 1,838 cases. In the Grand Anse and Sud Departments, the number of malaria cases was greater in January than during other months; in the Sud-Est Department, the peak number of cases was reported in April.

### Health facility exclusion.

Although all health facilities with confirmed malaria cases in the primary and secondary coverage areas were eligible for inclusion (*N* = 84; Figure 1), two refused to participate, and there were logistical constraints accessing 25 facilities. For the 27 facilities not visited because of refusal and logistical constraints (excluded areas in black in Figure 2A), the yearly total number of positive malaria cases recorded in the HSIS was 411 cases and the yearly median number of cases per site was seven cases (range: 1–65 cases).

### Spatial distribution of cases.

The spatial distribution of cases’ households was compared with a completely random spatial distribution using the Global Moran’s I test (Table 2). Clustering was present in two of 10 coverage areas: coverage area 1, Dame Marie and Anse d’Hainault (*P* < 0.01), and coverage area 2, Jérémie and Roseaux (*P* < 0.01). The average distance of the nearest neighboring household with a malaria case varied by area (range: 78–1,040 m). In nine areas, significant clustering was observed, indicating that households with malaria cases were, on average, < 1 km apart (Table 2). The Incremental Global Moran’s I test found peaks of significant clustering in Dame Marie and Anse d’Hainault, at 730 and 930 m, with two tighter peaks in Jérémie and Roseaux, at 150 and 270 m.

**Table 2:** Household-level spatial analysis of distribution of malaria cases

No.	Coverage areas	Households (*N*)	Observed average nearest neighbor (m)	Global Moran’s Index	Incremental Global Moran’s Index peaks (m)
1	Dame Marie and Anse d’Hainault	346	87.1*	0.07*	730*, 930*
2	Les Irois	39	82.5*	–0.12	None
3	Chambellan and Moron	13	228.3*	–0.05	None
4	Jérémie and Roseaux	343	77.9*	0.07*	150*, 270*
5	Bonbon	9	91.0	n/a†	n/a
6	Les Anglais, Chardonnieres, Port-a-Piment, and Coteaux	124	110.2*	–0.08	None
7	Torbeck, Les Cayes, and Cavaillon	67	785.4*	–0.014	n/a
8	St. Louis du Sud and Aquin	28	1,040.3*	n/a	n/a
9	La Vallée du Jacmel and Jacmel	162	267.5*	0.04	None
10	Cayes-Jacmel, Marigot, and Belle Anse	15	904.7*	n/a	n/a

* Significant at the 99% confidence level.

† Four areas where too few cases were geo-located to conduct reliable analyses of households.

### Adjusted cumulative incidence rates.

**Figure 4.**
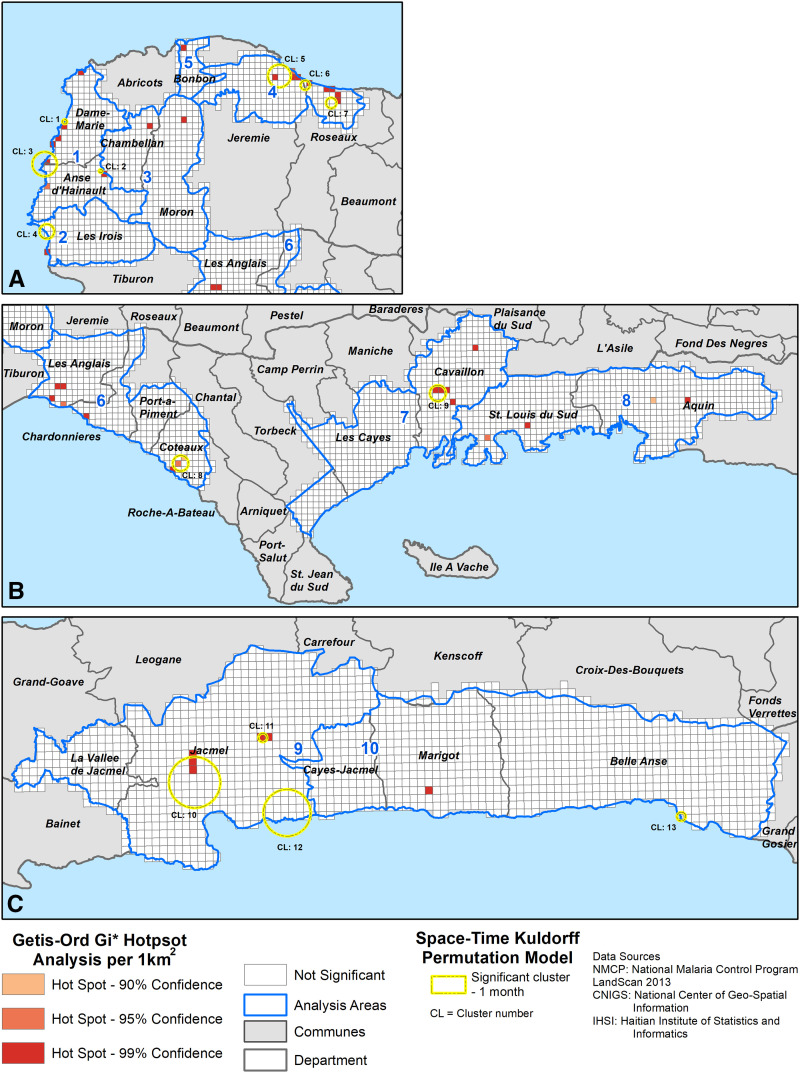
Getis-Ord Gi* cluster analysis of malaria cumulative incidence rates and 1-month Kulldorff space–time permutation clusters. Forty-three malaria incidence rate clusters were identified using Getis-Ord Gi* and 13 space–time clusters were identified from October 2014–September 2015. (**A**) Twenty Getis-Ord Gi* clusters and seven space–time clusters (*P* < 0.05) were detected in the Grande Anse Department. (**B**) Seventeen Getis-Ord GI* clusters (15 clusters at *P* < 0.05, two clusters at *P* < 0.10) and two space–time clusters (*P* < 0.05) were detected in the Sud Department. (**C**) Six Getis-Ord Gi* clusters and four space–time clusters (*P* < 0.05) were detected in the Sud-Est Department. This figure appears in color at www.ajtmh.org.

Adjusted CIRs were calculated for each 1-km^2^ cell (Figure 2B–D). Incidence rates are shown by shaded cells with levels from one to 1,000 per 1,000 persons; cells in white have a malaria incidence of zero geo-located malaria cases per 1,000 persons. Of the 249 cells with CIRs greater than zero, the average CIR was 99 cases per 1,000 persons (IQR: 50.45); 11 cells had CIRs at 1,000 cases per 1,000 persons. The Getis Ord-Gi* method identified 43 hotspots, each 1 km^2^ (*P* < 0.05), primarily in coastal areas in Grande Anse and inland areas in Sud and Sud-Est (Figure 4).

### Space–time permutation models.

In total, 13 unique statistically significant (*P* < 0.05) space–time case clusters were detected with an average radius of 1.24 km (172 cases) (Figure 4). Clusters in Dame Marie (CL3) and in Jacmel (CL10) had the greatest number of cases, with 47 and 45 malaria cases, respectively. Four clusters contained more than 10 cases per cluster. Most space–time clusters detected overlapped with the Getis-Ord Gi* CIR hotspots; however, three clusters (CL4, CL12, and CL13) did not overlap.

In the Grande Anse Department, five space–time permutation clusters were detected along the coast overlapping with six Getis Ord Gi* clusters. The space–time cluster with the greatest number of cases (*N* = 47) was identified spanning the neighboring communes of Dame Marie and Anse d’Hainault (CL3). In the Sud Department, space–time clusters had small numbers of cases (*N* = 4–5). The coastal pattern observed in other departments was different in the Sud-Est Department, with two noncoastal clusters (CL10 and CL11) detected in Jacmel with high cases (*N* = 22 and 45).

## DISCUSSION

The NMCP successfully launched a pilot GIS enhanced surveillance system in three departments covering 59 health facilities. In 1 year, 2,462 laboratory-confirmed malaria cases seen at these health facilities were identified and line listed retrospectively, and 58% of these cases were found at their residences. This system required relatively minimal human resources, consisting of four GIS assistants and one GIS analyst, each with university training, to cover priority areas. The system was designed to integrate into the existing passive surveillance system and to collect complementary data. This is the first time Haiti’s NMCP routinely collected health facility information and household locations to identify malaria case clusters at a fine scale.

Clusters were detected at multiple timescales, which provided the NMCP with specific months and locations to consider for targeting future intervention strategies. The malaria CIRs show many areas with high incidence that may be discrete high-transmission hotspots. Kulldorff space–time permutation clusters that were not detected when using malaria CIRs suggest unstable clusters that may be driven by factors such as asymptomatic carriers and changes in vector density.^7^ Or, these clusters could represent stable transmission hotspots with too few cases to be identified using malaria CIRs or they were falsely detected clusters. The space–time clusters that overlapped with Getis-Ord Gi* clusters may be stable clusters. The duration of clusters and completeness of cases geo-located from the Sud-Est Department indicates that these are more likely stable hotspots that warrant further investigation and action.

The Getis-Ord Gi* clusters detected could be used to guide programmatic investigations and to conduct targeted foci interventions. Overlapping clusters identified using both Getis-Ord Gi* and Kulldorff space–time permutation models could be prioritized, because these are likely to be stable clusters. For example, the NMCP’s vector control field workers could conduct entomological assessments of identified hotspots to assess mosquito habitat dynamics (e.g., permanence, positivity), treat or eliminate prolific larval development sites (i.e., concentrated, fixed, and accessible), and classify the focus as active, cleared, or nonactive.^6^ In addition, the NMCP could investigate why there are more cases in these clusters by assessing geographic accessibility to health facilities, timeliness of treatment, migration patterns, and other factors related to health-seeking behaviors. More data from community-level investigations collected across multiple years in these clusters, and in areas not identified through these methods, could assist the NMCP in understanding how well the spatial methods reflect the underlying malaria distribution. These validation objectives outlined in supporting research were beyond the scope of this pilot program.^5,17^

### Limitations.

Operationally, the pilot program areas were too large geographically to cover routinely with the project resources. In addition, there were delays in the launch and implementation, such as delayed distribution of funds and a staffing gap of one GIS assistant. As a result, the workload assigned per GIS assistant was too high. Therefore, because GIS assistants prioritized visiting health facilities and communities closer to their residence, potential bias was introduced that field-located cases were more likely to be identified along roads and near their cities of residence. Clusters in remote, hard-to-reach areas were less likely to be detected through this pilot program.

It was difficult to verify line-listed cases against aggregate health facility data (duplicate data in HSIS) or against the MMR only (incomplete data) because malaria test results were often recorded only in one register. If health facilities that diagnose patients with both microscopy and RDTs record positive tests from both procedures of the same patient on the same date, this usually resulted in two malaria cases being reported in the HSIS. Because of this practice, in 2014 and 2015, the total number of malaria cases reported at the aggregate level was slightly overestimated (10–85%) for four facilities. In addition, MMRs often did not include patients seen on the weekends, patients from the emergency room, and patients who went directly to the laboratory to avoid a registration fee. If the confirmed malaria case was not recorded in the MMR, it was not possible to locate the patients’ medical record or household. These challenges might have contributed to incomplete capture of malaria cases in the HSIS.

Although the maximum cluster size searched for in the space–time analyses was 3 km (based on the average flight distance of mosquitoes), people do travel greater distances. An increase in the maximum cluster size to account for daily travel may increase or decrease the number of clusters and cluster size. However, the evening and nighttime biting habits of *Anopheles* and the low reported overnight travel to other communes from interviews (< 1%) suggest that the identified clusters are associated more with localized transmission rather than with travel to other communes.^1^

This pilot system was not designed to test or identify additional asymptomatic or symptomatic individuals. These analyses only represent individuals who sought care at health facilities, were confirmed with malaria by RDT or microscopy, and were geo-located between October 2014 and September 2015. Studies show that a small proportion of asymptomatic infections are reservoirs for continued transmission in communities.^6^ It is likely that Haiti’s clusters, which were detected retrospectively using symptomatic patients, include asymptomatic individuals who may be contributing to ongoing malaria transmission. In a cross-sectional health facility survey, Elbadry et al.^18^ found that the asymptomatic infected population varied between the Departments of Grande Anse and Sud-Est, 40.7% and 21.6% positive, respectively. The testing method used was reverse transcriptase–polymerase chain reaction (RT-PCR), which resulted in a greater number of positives in their sample than either RDT or microscopy.^18^ However, it is unknown whether RT-PCR would identify different clusters than those identified using RDT and microscopy.

It is not known whether testing for asymptomatic infections at the health facilities and monitoring confirmed positives would lead to identifying additional high-transmission hotspots or would result in a better programmatic outcome. Because stable hotspots are usually detected across multiple years, some studies have incorporated serological testing for exposure to *P. falciparum*, which provides information on previous malaria infections.^19^ The addition of serological testing may aid in the identification of stable hotspots across time. Although beyond the pilot’s scope, a current malaria serosurvey may provide for additional validation and improvement of the models. Nonetheless, Haiti’s pilot approach to identifying clusters serves as a feasible first step to identifying hotspots where further foci investigations and malaria control interventions can be targeted.

### Recommendations.

Moving forward, as the NMCP transitions to a national malaria case-based surveillance system, the following recommendations can improve the implementation and integration of the geo-location of cases’ households as a routine activity. Departmental malaria coordinators, health facility directors, archivists, and laboratory technicians are key stakeholders to include in implementation. As a result of the pilot, health facility staff began recording communal section, village, and address of cases’ households to improve data quality. When all three fields were recorded, 85% of households (*N* = 859 of 1,012 households) were located successfully. Pilot recommendations for strengthening surveillance include 1) reviewing all four registers, 2) recording all confirmed malaria patients’ residence information on intake, and 3) documenting an adult (> 18 years) for each minor patient.

As malaria case management and reporting are expanded to community health workers (CHWs), CHWs could record malaria test results and GPS coordinates of patients’ residences, thereby greatly enhancing the system’s efficiency. We recommend identified hotspots be examined further by the NMCP’s entomological and epidemiology teams, and upon examination, for teams to take appropriate control measures outlined in Haiti’s national strategic response plan to prevent transmission to more households.

## CONCLUSION

The NMCP’s GIS enhanced surveillance pilot used geo-location successfully for 58% of 2,462 malaria cases from health facility registers to detect 43 hotspots with 1-km^2^ areas and 13 space–time clusters in relatively high- (Grande Anse), medium- (Sud), and low- (Sud-Est) risk transmission departments. Despite operational difficulties, the results of this pilot program provided crucial information for Haiti. This system is already informing future elimination strategies and resources needed for scaled-up activities.^20^ The NMCP used these data to prioritize areas to add CHWs. In 2017, as the NMCP began transitioning to facility-level case-based malaria reporting, surveillance training highlighted the review of all four registries and completion of three address fields. This GIS enhanced surveillance system is an example for other countries with low or very low malaria transmission that are transitioning from collecting monthly aggregated data to individual patient data.^5^ Additional entomological assessments and household-level surveys would provide ideal opportunities to evaluate and improve further the use of these geospatial methods.
